# Guiding Mothers About Early Detection and Addressing Speech Delay and Disorders Among Children in a Rural Setup

**DOI:** 10.7759/cureus.48822

**Published:** 2023-11-15

**Authors:** Akshaya Narasimman, Sunita Vagha, Akshat K Kashyap

**Affiliations:** 1 Pathology, Jawaharlal Nehru Medical College, Datta Meghe Institute of Higher Education and Research, Wardha, IND

**Keywords:** assessment of mothers, treatment for speech delay, language skills, maternal awareness, delayed milestone, speech disorders

## Abstract

Introduction

Speech is one of the most important milestones to be achieved by a growing child. The significance of being informed about different pediatric speech abnormalities, especially to mothers, allows them to help their children in cases of irregularities in the maturation in this domain.

Aim and objectives

The study aimed to assess and educate mothers on the important milestones of speech delay in children and make them address the issue and be aware of various corrective measures to treat the underlying conditions of speech disorders in children. The objectives of the study include understanding the pre-acquired knowledge of the mothers regarding the delay in speech in children, imparting knowledge regarding different speech disorders and their management, spreading awareness on how to seek help for various underlying causes of speech irregularities or delay, and to train the mothers into approaching the challenges in an orderly manner.

Methods

A study was conducted to guide rural mothers visiting the Obstetrics and Gynecology and Pediatric out-patient departments and Neonatal wards in a rural tertiary care hospital situated in the Sawangi Meghe village of Wardha City, Maharashtra, India, about the detection and treatment of children with disabilities of speech as early as possible. The mothers' knowledge was assessed before and after the study with the help of questionnaires, and basic understandable information on different types, causes, symptomatology, and management of speech delay and disorders among children was explained with the help of group discussions and posters.

Results

The motive behind this study was to be aware of facts known by the rural mothers, their actions on coming across such presentations by their children, cues that they would pick up, and the need to ask for help at the appropriate time were assessed and elaborated if not known by them. The Relative Learning Gain and Normalized Gain were calculated to be 76.43% and 0.74 (high gain), respectively, and out of the total subjects, 97.16% of mothers voted that this study proved helpful, and six mothers (4.23%) benefited with the intervention and were referred to experts for evaluation of their children.

Conclusion

Awareness in this field is necessary to manage children's development, especially by their mothers. Knowing the prevalence of knowledge in mothers may build an association with the prevalence of the recognized cases of speech disorders in children. Evaluation at different community levels may be conducted to gauge the need to impart required knowledge about speech disabilities in children to the maternal population. Future research and the impartation of knowledge to caregivers are vital to promote vigilant and systematic action to be taken regarding the proper growth of their children.

## Introduction

Problem statement

Speech is a vocalized mode of expression that involves the basic four functions of producing speech which are initiation, phonation, oro-nasal process, and articulation. When a child's natural speech pattern is incomprehensible or characterized by phonetic mistake patterns that are inappropriate for the child's age, speech delay may be a developmental problem. According to estimates, 40%-60% of kids have improper speech and language delays, which increases their chance of developing socio-psychological, behavioral, and cognitive problems as adults [1]. A child begins to communicate in monosyllables between the ages of one and three years, and by four years of age, they can compose complete words and phrases. Although some kids may be slow communicators, the delay could also indicate a more significant problem, including global developmental delay, which would call for a thorough evaluation. Various ante-natal, post-natal, and external factors may cause such developmental delays, and therefore, knowledge and awareness of the parents are of utmost necessity to identify and recognize any signs of the need for interventions [2,3]. There is a cascade of interrelated processes at various stages of speech creation. The most common forms of speech and language delay are language comprehension and reception disorders, vocal cord malformations, and tongue and trauma-related speech abnormalities. The causes of speech delay may be hearing loss, neurological disability, autism, physical speaking problems, or selective mutism [4].

When the speech development milestone is missed, it can lead to maternal depression in the first few years after giving birth and may even last longer if the disease is not treated and diagnosed at an early stage [5]. Studies have established that maternal traits, including the mother's age, education level, and status in the workplace, are related to children's language development and call for social and educational interventions [6].

Background

The WHO's (ICD-10) classification of speech developmental issues includes articulation deficiency, expressive speech delay, and remarkable speech. Verbal therapy approaches for articulation issues, stuttering, and poor expressive speech have shown the best results in improving the quality of life of children [7]. For the treatment of speech delay and disorders, speech pathologists' consultations, articulation drills, motor learning through repetition of the tongue movements, and coordination of the lips and jaw necessary for accurate pronunciation, pharmacological approaches, and appropriate counseling may prove to be effective [8]. An observational type of study with proper interventional strategies is designed in such a way as to retrieve data from the required section of the population at two points with a sensitization session in between them. These cross-sectional studies are achieved by using pre-awareness and post-awareness forms to evaluate the amount of awareness they had before the study and the relevance of the intervention made by analyzing the results after the awareness. These studies can associate the intervention with the revised outcome of any study [9].

Relevance

The relevance of this study in a rural setting was to educate the mothers with the help of posters, group discussions, and enactments to have a significant impact on the understanding level of the mothers about speech disorders in children.

A thorough overview of the child's health, family history, aptitude, and rate of development should be done in tandem with cognitive function testing while managing a child's speech pathology. To help the child have a positive social, emotional, and psychological childhood and adulthood, the mother, other family members, and consulting specialists must all be informed of the difficulties the child is experiencing [10].

Rationale

Speech delay and disorders in children are associated with a wide range of factors that affect the ability to speak in a child. The mother is the constant caregiver and carries a tremendous responsibility of acquainting herself with the knowledge regarding the developmental milestones of speech in her children. The studies conducted on the linked factors and prevalence varies broadly as the criteria of these analysis included different kinds of age groups, methodologies, and perspectives in their studies. There have been minimal studies evaluating the knowledge of mothers, especially in India, which, being a developing country, has a larger section of the rural population who have minimum information about the management and care for speech-disabled children [11].

Studies have associated several environmental and biological factors together, along with the socio-economic status affecting the language development of the child. Therefore, the rationale behind this study was early detection of such anomalies which greatly assist in preventing and treating children to reduce the long-term effects of in the future. Thus, the current study focuses on imparting relevant information to the mothers as maternal education is necessary to be vigilant in such situations so as to reduce stress and anxiety in them and the family [12,13].

Significance

The significance of the study was to improve the pre-existing knowledge of the mothers falling under the criteria of the study to take the basic steps in asking for the right professional help in case they notice a delay or an abnormality in the speech of their child. A normal, healthy infant develops early speaking and language skills between the age group of eight months to three years and the earliest way of interacting is by crying, babbling, and gesturing, which slowly progresses into putting together words and forming short sentences explained to the mothers. Early interventions by the parents in case of speech disorders, should include talking, listening, observing the child's behavior in day-to-day activities, and easing the child to try and learn the language and articulation of the words. Criticizing, interrupting, and correcting the child frequently may lead to further distress and inhibition of language comprehension. The child should be counseled and consulted with a speech-language pathologist (SLP), pediatrician, psychologist, or audiologist in case of reduced response to the continuation of the difficulty in speaking [14].

## Materials and methods

This is an interventional type of cross-sectional study with a study duration of two months and included mothers from Obstetrics and Gynecology OPD, Pediatrics OPD, and neonatal ward who visited the departments in a rural tertiary care hospital. Proper consent was taken from the mothers interested in the awareness; the mothers were well-informed about the study's methodology, and the ultimate aim of the study was elaborated to them. The intervention was done with the help of posters, photographic representations, PowerPoint presentations, short discussions, enactments, and an overall interactive session to evaluate and educate regarding the study. This method used in the intervention where consistent with the cultural norms and beliefs of the rural community participating in the study.

Methods of intervention

Inclusion Criteria

It included different categories of mothers having children with or without speech irregularities/delay, antenatal mothers, others having toddlers (zero to three years), mothers with children aged more than three years, and mothers having adolescent children.

Exclusion Criteria

It included mothers who have crossed the milestone of speech development in their children.

Data Collection Instruments

It comprised the pre- and post-awareness questionnaires, which were circulated, and data was collected to assess their knowledge before and after the interventional study. The study was conducted among mothers visiting the Obstetrics and Gynecology OPD, Pediatrics OPD, and Neonatal ward of a rural Tertiary Care hospital in the Vidarbha Region of Maharashtra.

Sample Size

A total of 141 mothers fulfilled the study's inclusion criteria by applying the simple random sampling technique. A pilot study was conducted to check the reliability of the questionnaire prepared for the research on 15 subjects, which came to be >0.7, which proves the reliability of the questionnaire to be used in this original research study [15]. The validity of the questionnaire was accessed by five experts on the field with a Content Validity Index of 0.8857, and it was accordingly improved before using in the interventional study.

The sample size is based on the Krejcie and Morgan methodology of calculating sample size to simplify the pre-estimated population size of 220 mothers (average number of mothers coming to the OPD, fulfilling the inclusion criteria) in the limited time duration of two months, considering 50% of awareness acquired (population proportion) as given below [16]. $n = \frac{x^{2}*N*P*(1-P)}{ME^{2}*(N-1)+[x^{2}*P*(1-P)])}$Sample size n = 141 Where: n = sample size, N = Population size 220 (Average number of mothers falling in the inclusion criteria considering the feasibility of data collection in the duration of the study). X2 = Chi-square for the specified confidence level at 1 degree of freedom = 3.8416; P = population proportion=0.5; ME = desired Margin of Error (expressed as a proportion) = 0.05. Data analysis was conducted using the Statistical Package SPSS (version 27.0, IBM Corp., Armonk, NY), Microsoft Excel 2020 Version 1.0.1, and GraphPad Prism version 7.0.

Steps of intervention 

Step One: Accessing Awareness

This included evaluating awareness by assessing the mothers' pre-existing knowledge about the developmental delay of speech and language, its pathological aspect, complications, and control of various speech disorders. Pre-awareness questionnaire forms (attached in annexure section) were provided to be filled by the study population.

Step Two: Intervention

This consisted of guiding mothers about the manifestations of primary and secondary pathological conditions due to problems in different levels of speech development, its rightful addressal at an early age, and instructing them towards effective management. The interaction was through communication in Hindi and the vernacular language of Marathi, which helped build a rapport with the subjects and eased them into communicating and sharing their knowledge and experience about the theme and objective of this research study. Points covered under the sensitization process include the causes, symptomatology, the age at which the child starts to talk in monosyllables, approach to evaluation in such recognition of cues and importance of early detection of speech disorders. Intervention was done with the help of posters, photographic representations, power-point presentations, short discussions, enactments, and an overall interactive session in their native languages to evaluate and educate regarding the study.

Step Three: Follow-up and Feedback

This involved follow-up and feedback where the mothers in the study were asked to fill out a post-awareness questionnaire form (attached in the annexure section) for understanding and tabulating the information that would have reached the study population, which would help them take immediate and proper measures if such a concern arises concerning their children. Feedback was taken from them on the usefulness of the study.

Step Four: Effectiveness of the Intervention

It focused on assessing the effectiveness of the intervention, where the outcome of the awareness was analyzed with emphasis on the gap of knowledge filled by the initiative of the study, its benevolence to the target population, and its assistance in the diagnosis and ease of its control.

An informed consent form was given to the subjects to fill out after making them understand the study's objectives; confidentiality was maintained throughout the research, and information about the subjects was accessible by the researchers only. The study was conducted only after clearance from the Institutional Ethics Committee (IEC) of Datta Meghe Institute of Medical Sciences Wardha with approval number DMIMS(DU)/IEC/2022/85.

## Results

A total of 141 mothers coming to the Obstetrics and Gynecology OPD, Pediatrics OPD, and Neonatal wards were assessed who satisfied the study's inclusion criteria, as shown in Table 1.

**Table 1:** Different subjects satisfying the inclusion criteria of the study.

Inclusion Criteria of mothers	No. of subjects
Antenatal mothers	49
Mothers having toddlers	57
Mothers with children aged>3 years	26
Mothers having adolescent children (10 to 19)	9
Total subjects	141

**Figure 1:**
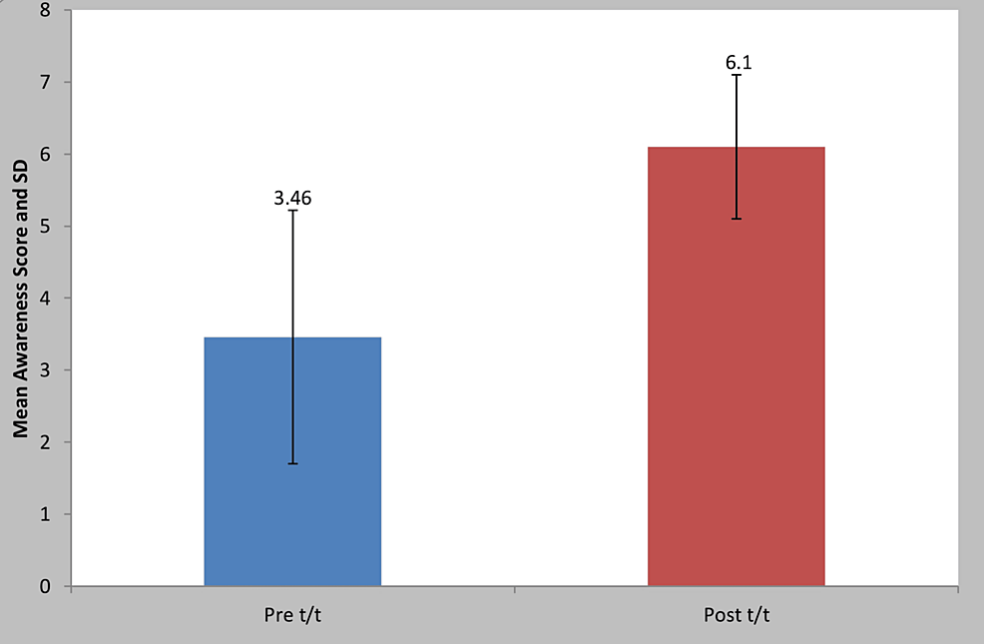
Comparison of awareness of mothers evaluation values at pre and post-awareness studies. This bar graph represents the mean awareness values and standard deviation pre- and post-test where p<0.05 is considered significant.

The pre-and post-awareness forms provided to the subjects had seven questions, each consisting of closed questions (yes or no) with a binary coding of one and zero, respectively. The codes are evaluated below in Table 2, with its pictorial depiction of the mean awareness values in Figure 1.

**Table 2:** Student's paired t-test. This table shows the mean, standard deviation, standard mean error, mean difference, t-value and p-value, where p<0.05 is considered significant.

	Mean	N	Std. Deviation	Std. Error Mean	Mean Difference	t-value
Pre t/t	3.46	141	1.76	0.14	2.64±1.59	19.63 P=0.0001,S
Post t/t	6.10	141	1.00	0.08		

**Figure 2:**
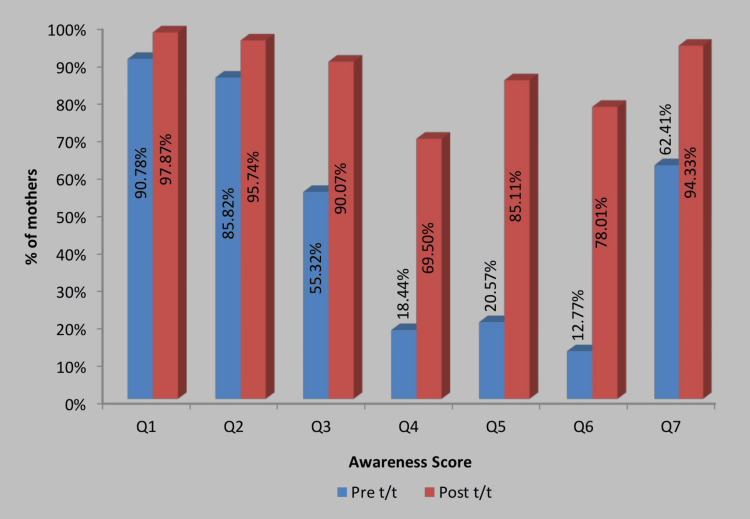
Question-wise comparison of awareness values of mothers at pre and post-test. This bar graph depicts the awareness among mothers based on the questionnaire provided to them before (in blue) and after (in red) the interventional study, where p<0.05 is considered significant.

At the end of the study, out of the 141 mothers who participated, 138 (97.87%)of them knew about the occurrence of speech disorders in children, 135 (95.74%) of them knew at what age a toddler starts to speak in monosyllables, 127 (90.07%) knew what cues to look for and in case of any development of speech irregularity, 98 (69.50%) knew different types of speech disorders, 120 (85.11%) of them knew what steps to take in management speech disorders, 110 (78.01%) of them knew the basic causes behind the speech delay/disorders and 133 (94.33%) knew to act as soon as possible to help the child in case of speaking disability. Out of the 141 participants in the study, 137 (97.16%) mothers voted that this study proved useful and caused no anxiety or stress in them. After the intervention was conducted, six subjects co-related with the disorders present and the associated speech delay or communication abnormalities in their children (4.23%) and were referred for expert evaluation for their speech abnormalities. A comparison of values before and after intervention is given in Table 3, with its pictorial representation in Figure 2.

**Table 3:** Item-wise comparison of awareness values of mothers pre and post-test. This table shows the awareness of mothers for each question provided to them in the questionnaire before and after the intervention was conducted along with the X2-value and p-value, where p<0.05 is considered significant.

	Pre t/t	Post t/t	Χ2-value	P-value
Q1	128(90.78%)	138(97.87%)	4.71	0.02,S
Q2	121(85.82%)	135(95.74%)	6.10	0.01,S
Q3	78(55.32%)	127(90.07%)	30.72	0.0001,S
Q4	26(18.44%)	98(69.50%)	54.87	0.0001,S
Q5	29(20.57%)	120(85.11%)	82.22	0.0001,S
Q6	18(12.77%)	110(78.01%)	85.19	0.0001,S
Q7	88(62.41%)	133(94.33%)	29.84	0.0001,S

Mean % of pre-test value came out to be 49.44±25.18; mean % of post-test value as 87.23±14.35; relative learning gain {[(% post-test) - (% pre-test)]/(% pre-test) ]× 100} to be 76.43%; absolute learning gain [(% post-test) - (% pre-test)] as 37.79 and normalized gain (g) [(% post-test-%pre-test)]/[(100-(%pre-test)] as 0.74 (high gain). The significant effect of the intervention was measured using the range of Normalized gain “g” with references of 0-0.29 depicting low gain, 0.30-0.69 as medium gain, and 0.70-1.0 as high gain. Statistical analysis was done by using descriptive and inferential statistics using the student's paired t-test and Chi-Square test. The software used in the analysis was SPSS 27.0 version (IBM Corp., Armonk, NY) and GraphPad Prism 7.0 version, and p<0.05 was considered the level of significance.

## Discussion

Speech disorders in children are multifactorial causations resulting in poor maturation and growth of the child. Many studies have found the evidential necessity to gain information about speech delays in children and to provide overall support to the speech-disabled child to attain a better quality of living and develop proper speech abilities. Parents are the closest caregivers, and their responsibility to identify and raise red flags if milestones are missed in the developmental process is paramount; therefore, awareness of experiencing the power of knowledge is necessary to know the significance of meeting milestones in a growing child. In this study, the Relative Learning Gain, Absolute Learning Gain, and Normalized Gain were calculated to be 76.43%, 37.79 and 0.74, respectively, which signifies the successful implementation of the interventional process and understanding of the mothers of the basics of language and speech diseases. Evaluation of the awareness forms and the analytical approach associate the meaningful impartation of knowledge and its utility to the mothers. Other studies that have been conducted focus on the incidence of different speech delays and irregularities in children, the association of different risk factors, and environmental considerations. These become the basis of interventional studies to spread the calculated and latest evidential data to subjects to educate them about speech-related disorders.

Analysis of this study shows that the maternal population is unaware of three things: different types of speech disorders (79.43%), which factors may cause speech delays (87.23%), and what are the ways to manage and provide medical treatment for the child (37.59%). The importance of early detection and address of such complications are known to 62.41% of mothers before the intervention, which may also be a result of concern the mothers have in case of any difficulty faced by the child. The post-awareness data had a significant rise in the percentage of mothers becoming aware of the depth of the condition and the course of action to be taken in such circumstances. Research studies and systematic reviews on various speech disorders give us the information to consider the possibilities of their occurrences and know the risk factors related to it for easy and early identification of childhood speech disorders. These benefit this study to educate the mothers about the risk factors, symptoms shown, and the available management therapies. Table 4 shows different causes affecting speech delay and disorders and Table 5 depicts normal age-group-specific cues by which speech development may be assessed as normal or impaired, as explained to mothers in the intervention.

**Table 4:** Different types and causes of speech delay and disorders seen in children. FOXP2 - Forkhead Box Protein P2

Serial no.	Cause of speech delay/defect	Type of speech defect/disorders
1)	Neurological	Autism Spectrum Disorder, Childhood Apraxia of speech, Dysarthria, Attention Deficit Hyperactivity Disorder, cerebral palsy
2)	Structural defects	Cleft lip and palate, orofacial and myofunctional deformities, tongue deformities, breathing/airway problems, vocal cord disorders
3)	Hearing impairment	Hearing loss, ear infections (otitis media)
4)	Phonological	Defects in language speech sound delivery, speech sound disorders, stuttering
5)	Articulation defects	Distortion in the production of individual speech sounds
6)	Physical trauma/head injury	Right Hemisphere injury, traumatic brain injury
7)	Psychological	Traumatic childhood experience, fear
8)	Environmental	Bilingualism
9)	Genetic causes	Down’s syndrome, FOXP2 gene mutation, etc
10)	Ante-natal and perinatal factors	Maternal addictions, antenatal trauma or complications, low birth weight

**Table 5:** Different age groups in co-relation to speech impairment cues seen in children.

	Age-group	Signs of language/speech impairment
1.	4-7 months	Crying, cooing, and babbling sounds are less, abnormal, or absent
2.	7-10 months	Early gestures like pointing, and making eye-contact while trying to vocalise are absent
3.	12-15 months	Unable to say small or few words or make sounds
4.	15-18 months	Does not understand words and is not able to communicate by words or sounds
5.	2-2.5 years	Has difficulty while playing with same-age peers, forming small sentences
6.	2.5-3 years and above	Has difficulty in comprehension, reception and expression of thoughts, words and sentences

Dysfluency in speech or stuttering in children is recognized as repetition of words, prolonged pauses between words or sentences, and loud extension of words while communicating accompanied by involuntary blinking and head or jaw movements. Researchers studied that genetics usually determines this and involves neurological deficits more than psychological problems. Speech therapy is known to be very critical in such cases as there are chances of it getting resolved in adulthood. The presentations of stuttering were enacted and explained to the subjects in the awareness study for them to be able to recognize such cues in case of developmental disorders in their children [17].

Studies have been conducted on the Childhood Apraxia of speech, in which children have difficulty pronouncing sounds and syllables coherently and accurately. Pronunciation of words and sentences clearly and in rhythm is affected due to neurological defects in the speech centers and muscles involved in sound production. Misarticulations and omissions, limited or no babbling in infants, oral “groping” - where the mouth is opened, and lips move but there is no speech and monotonous speech production are the cues that mothers could recognize in detecting a speech problem in the child. Different assessment tools used to diagnose this condition and treat it correctly were studied. Thus, the study backs the information on available diagnosis and treatment of children provided in the awareness procedure in the current study [18,19].

Attention deficit hyperactivity disorder (ADHD) in pre-term and term infants harms the child's long-term speech development and language understanding. The study associated the delay with maternal habits and comorbidities. Hyperactive behavior coupled with lack of concentration and speech delay indicates ADHD, especially in two- to five-year-old children. These help this study to understand and make mothers observant of their health and their child's and to pick up the proper cues of abnormal speech [20,21]. Autism spectrum disorder (ASD) in children also requires interventional techniques of language and speech therapies to improve social interactions and academic achievements in the child [22]. Thus, awareness of different aspects impacting children's speech development is required, which aligns with the objective of the current study.

Cleft lip, cleft palate, and other cranio-maxillary deformities have been common defects that are presented congenitally, and researchers have studied the need to understand the behavior and signs in children who are unable to speak need is very important in the closest caregivers to provide the children with the care and attention they deserve. The study indicated surgical treatment as a method that could restore normalcy and a reasonable standard of living in the child. The present study also aimed at combating unfavorable attitudes and ideas and the importance of having enough knowledge and understanding about the causes, treatments, and prevention, as parents' knowledge may encourage better health in their wards [23,24].

Researchers studied bilingualism and its attribution to the slow rate of language learning and speech delays, which needs to be re-shaped with scientific evidence indicating that monolinguals and bilinguals are equally susceptible to developmental delays. The current study also focused on eliminating the misunderstanding of the caregivers by advising them to expose their children to both languages for significant periods, as children can learn more than one language efficiently, especially in the early stages of their lives [25].

Hearing loss in infants and children is frequently persistent and debilitating, influencing the development of speech and language, cognitive function, intelligence, emotions, and social-cultural status in society. Research on the early screening of hearing impairment for rehabilitation and the necessity to fill the gap in mothers' knowledge and its association with communication delays for the betterment of the child's future was studied. Negligence to hearing loss in children may lead to poor speaking skills, which was covered in the present awareness study [26].

Research on mothers with children having cerebral palsy has increased anxiety and stress associated with the degree of the severity of the disease of their children. Low family support and less awareness of various methods to cope and deal with the distress led to a lower quality of living and sociological difficulties in the mothers [27]. Studies associated traumatic brain injury in children to be mainly caused by road traffic accidents or physical head trauma to the baby due to falling or slipping. These were concluded to be prevented by being cautious and quick in getting medical aid; psychological trauma in childhood is also a reason for late speech development. These studies give a very important point of awareness to the intervention provided to the mothers, as done in the present study, to give the infants and the growing children the utmost care and medical treatments as early as possible because there may be developmental disorders if not managed in time. Knowing how to deal with extreme developmental abnormalities in their children may even reduce stress and anxiety in mothers [28,29].

Research studies have been conducted on the genetic mutations of genes related to speech and language development, like the FOXP2 gene mutation and its close relation to speech and language delay. The outcome indicated that prenatal genetic testing could be done with a family history of any speaking disorders to diagnose the speech or language delay that may manifest in the child due to the gene mutation [30]. Down's syndrome is also a congenital chromosomal anomaly that leads to reduced craniofacial and neurodevelopment, resulting in language and intelligence limitations. Proper interventional methods are required to be provided by the clinicians and the family to such children to reduce the effect of the abnormalities. Education about genetic testing in antenatal mothers and its benefits was also explained in the present study [31].

The best way to maximize the benefits of early intervention, which leads to more effective communication outcomes in later life, is through early detection of speech and language problems. It has been discovered that learning and interpersonal communications due to speech and language impairments and disorders can persist throughout puberty and beyond if symptoms are not treated. A study conducted in 2008, focused on the early recognition of children with developmental disorders or delays, which may lead to intervention at an early age when possibilities for improvement could be higher. The causes, consequences, and interventional treatment methods were also studied to help diagnose the child by its early presenting characteristics and behavior, which indicates the need for awareness studies like the current study to educate mothers regarding changes seen in speech-disabled children [32].

Interventions provided to the children include group or individual therapy with assessments of language, phonology, articulation, and expression of language and comprehension. A study to know the efficiency of interventions provided to children with speech and language disorders was conducted, which showed a positive outcome. Therefore, it is vital to encourage parents and guardians to enroll their children for better future opportunities and academic achievements, which was one of the objectives of the present study [33].

All the existing research studies on the different types of risk factors and causes of under-developed speech in children point towards a better understanding of the various disorders in detail to manage the diseases systematically. Cross-sectional studies to know the prevalence and incidence in the population would be more efficient if the awareness of such studies reaches the population susceptible to facing such circumstances, especially the primary caregivers of children. Therefore, awareness studies are the need of the hour, especially in India, where the rural population still needs expertise and information regarding pertinent aspects of milestones in the development of children and the role of the mother in the process.

A study to know the knowledge possessed by caregivers about Language disorders in children, the interventions provided by SLPs, the clarity of their understanding regarding the disorders, and the procedures for managing it by the SLPs were carried out. The outcomes of the interviews indicated anxiety and confusion states of mothers with little awareness and trust in their SLPs in providing the right course of information about the diagnosis and management therapies for their speech-disabled children. The present study was also focused on knowing mothers' knowledge about speech disorders in children and assessing awareness given to them on the importance of understanding the child for their proper development. The study was limited to the knowledge of speech disorders. It did not include the perception and ideas of caregivers on SLPs and the interventional therapies given to children. Similarities in the outcome of both studies indicate the poor knowledge of the mothers about the subject [34].

A study in Taiwan was conducted to determine the association and relevance of concerns raised by the parents and the diagnosis of a developmental delay in the children. The preliminary worries of the parents on the child's cognitive, speech/language, emotional/behavioral, motor, and overall development showed little agreement with the outcomes of professional identification of the problem. The study's findings show that caregivers are concerned about a child's cognitive, speech/language, emotional/behavioral, and motor development. Still, it is important to ask whether these concerns are attributable to the issue. This indicates the need for studies such as the current research study, which aimed at spreading awareness about the cues and signs to identify manifestations of speech disorder in children for alignment with the diagnosis of any developmental delay by the clinicians for early detection and address of the problems [35].

An extensive scoping review of the pre-term communication skills and the mother's perceptions was carried out. Caregivers of pre-term infants encounter a range of challenging feelings and realities, and preterm infants are at risk of speech abnormalities or delays. The study's conclusions suggest the provision of suitable maternal assistance to prevent or treat these communication issues successfully. Health practitioners also should thus have a thorough understanding of mothers' impressions of early communication, interaction, and development in the preterm population. Only a limited amount of study has been done on mothers' perspectives of preterm infants' early communication development, which results in challenging feelings and emotions while being hospitalized and providing for their infants. The knowledge mothers possess regarding the child's communication abilities, the difference in normal and atypical growth processes, and normal ages of speech milestones are fundamental to assess and improve upon. Therefore, this study assessed the mothers' perceptions of communication skills and their disorders in children not limited to mothers of pre-term infants but included antenatal and post-natal mothers [36].

Yet another study to align the understanding of caregivers and SLPs was carried out which focused on the developmental consequences that may be impacted by caregivers' impressions of their child's language impairment, which may affect their participation in therapy and day-to-day interactions. The study's findings indicated caregivers' opinions were more favorable and varied than SLPs. However, SLPs and caregivers both agreed that perceptions of current and future results were more important than either competence or quality. A SLP could rely on the mother about the different symptoms like behavioral, academic performance, and emotional responses. Therefore, awareness studies focusing on identifying signs in children indicating disorders in speaking abilities should be conducted, similar to the current study [37].

Another such study to know the reasons for poor follow-up and general view among caregivers in case of discontinuity in the interventional processes was conducted which was based on the vitality of identifying developmental delays in newborns, toddlers, and young children as soon as possible and the caregiver's knowledge of the importance of regular interventional treatments. Children who are at risk of developmental delays can get appropriate intervention if they are identified early. Early intervention can decrease or eliminate the detrimental effects of a handicap on a child's development. Early intervention lessens the burden on the child, family, and society while favorably influencing a kid's development, behavior, and academic achievement. The study showed participants who neglected to follow up on referrals did so due to work obligations, practical difficulties, other commitments, and forgetfulness. After a developmental screening, enhancing follow-up compliance for early intervention is challenging and needs more study. The study at hand increases the significance of early detection from the perspective of the mothers, which may lead to them prioritizing the interventional therapy sessions of their children [38].

A study was performed to assess the factors that affect adherence to early treatment after diagnosis and screening of developmental problems in children by caregivers. The caregivers of children recommended for speech-language and occupational therapy filled out a risk assessment form. There was poor follow-up compliance for early detection programs, and major factors influencing were employment, logistical problems, other commitments, and ignorance. The study concluded that it is necessary to raise people's awareness of it and educate people about the value of growth for the better academic performance of their children, similar to the goal of the present study [39].

In a four-week interventional study of caregivers and their children, eight toddlers between the ages of 21 and 45 months were evaluated at four research visits, where validated tests were taken to gauge caregiver engagement and knowledge, language development, and autistic symptomatology. Analysis indicates that the intervention was effective, and social communication, language comprehension, and verbal fluency all improved along with caregivers' knowledge and subjective sense of engagement with their kids. This study shares a similar idea with the current study to acquaint the mothers with the basic idea of the common speech disorders associated with symptomatology for better understanding and communication to help develop and improve speech abilities in children [40].

A study was carried out to assess Iranian parents' knowledge of the idea and significance of early childhood development (ECD) and to identify the sources of that knowledge from the viewpoint of both parents and grandparents. The findings show that Iranian parents' awareness of ECD needs to be improved; hence, steps must be taken to improve their knowledge in these areas. Parents seek out trustworthy and legitimate sources to further their education and frequently turn to pediatricians. Therefore, it is advised that additional research be done on measuring parents' knowledge in the community and practical strategies for knowledge promotion in this area, which correlates with the objectives of the current study [41].

The present study fulfilled the necessity to educate rural mothers about developmental speech anomalies and their management, but it also raises a need to increase such awareness studies, which may focus on the different aspects of the emotional status of a mother with a speech-disabled child, the effect of socio-economic background on an autistic child or a child with a congenital speech defect and so on which may again call for interventional studies of awareness to maintain a strong mental, physical and social wellbeing, equally of the child and mother.

Limitations of the current study may include the language barriers and lack of education of the rural population, which may have led to difficulties in comprehending the intervention. Poor socio-economic status and less accessibility and guidance about such health-related issues and their treatment modalities may also affect their lack of knowledge and vigilance. The lack of a larger sample size due to duration constraints may also have led to a lesser understanding of the knowledge of the subjects and the effectiveness of the intervention provided. Ignorance among the subjects may also be a consequence of the normal developmental process achieved by their children.

The scope of this study limits itself to the observation of the understanding of rural mothers and the analysis of the effect that an interventional study would have on them. The impact proved useful with a significant learning gain, which expanded the scope of the study beyond the process of creating awareness. This study can motivate researchers to conduct more interventional and cross-sectional studies on the incidence and prevalence of childhood speech disorders. This may be evident in proposing sensitization sessions to governmental and non-governmental organizations, especially mothers and primary caregivers. The perceptions, attitudes, and understanding of caregivers on the interventional therapies of speech-delayed children with positive and negative outcomes and the possible causes may also be an area for future research. Suppose the necessity for immediate action arises with cumulative analysis of evidential data from such studies. In that case, health policies can be proposed by the central health councils regarding speech therapy, proper treatment and care of delayed speech in children, and early detection to prevent prolonged development of the disorder and difficulties in future socialization endeavors among adolescents.

## Conclusions

The study concludes that there is a significant need for an increase in awareness about speech delay and associated diseases in children due to an evidential lack of knowledge among the primary caregivers of the children, especially in rural areas. This research satisfies the aim and objectives of the study and proved to be of utmost help to the mothers in sensitizing them toward the primary etiologies, approaches, and management behind different speech problems in children.

Limitations of the study include language barriers and low literacy levels of rural people, the evaluation of fathers or guardians who may be the primary caregivers in many households, and considering late talkers in some cultures as normal, which may delay recognition and management of such missed milestones. The outcome of this study might open up new avenues for research, keeping in mind the limitations and for creating a foundation for policymakers to organize health camps and screening sessions for rural children to educate and for early detection of speech disabilities. The accessibility of such knowledge should be increased to include the rural people, as they have less exposure due to factors such as illiteracy, language barriers, work limitations, poverty, ignorance, etc. The most effective way to educate the mother is by explaining the various conditions of delay in her antenatal/postnatal/pediatrician visits, with the collective efforts by social workers and health policymakers in enforcing access to healthcare with the help of telemedicine, using health applications which guide mothers to monitor and track the developmental milestones of their children especially in rural areas. This may be very helpful in the early diagnosis of any disabilities for a proper line of treatment.

## Appendices

Annexure no. 1

**Table 6:** Informed consent form given to participants interested in the study.

Field	Information
Reference ID	2022-02114
Title	Guiding mothers about early detection and addressing speech delay and disorders among children in a rural setup.
Respondent's Name	[Left blank for the participant to fill]
Age	[Left blank for the participant to fill]
Contact No	[Left blank for the participant to fill]
Address	[Left blank for the participant to fill]
Consent Request Message	I, Ms./Mr. _______, an undergraduate student, do request you to voluntarily participate in the study being conducted by me for research titled, ‘Guiding mothers about early detection and addressal of speech delay and disorders among children in a rural setup.’The information provided by you will be completely confidential, and no part of it shall be revealed in any form. The information will be used for study purposes only. Your participation is voluntary, and you can withdraw anytime during the study period. I (interviewer) may contact you again to complete the study. Signing the consent form will mean you are willing to participate in the study.
Date	[Left blank for the participant to fill]
Participant's Consent	This is my own decision to participate in the study, and I am willing to participate in the study, not being forced by anyone to participate in the study.
Participant's Signature	[Left blank for the participant to sign]

Annexure no. 2

**Table 7:** Pre-awareness questionnaire for the study.

Question	Response
1) Are you familiar with speech disorders among children?	Yes/No
2) Do you know the age at which a child starts to talk in monosyllables?	Yes/No
3) Do you know the cues that you could pick up that might suggest a speech disorder/developmental abnormality in your child?	Yes/No
4)Do you know the different kinds of speech disorders in children?	Yes/No
5)Do you know what steps to take if you face such a problem?	Yes/No
6)Do you know the basic causes of the common speech delays and disorders among children?	Yes/No
7)Do you know that such problems should be addressed soon?	Yes/No

**Table 8:** Post-awareness questionnaire for the study.

Question	Response
1) Are you familiar with speech disorders among children?	Yes/No
2) Do you know the age at which a child starts to talk in monosyllables?	Yes/No
3) Do you know the cues that you could pick up that might suggest a speech disorder/developmental abnormality in your child?	Yes/No
4)Do you know the different kinds of speech disorders in children?	Yes/No
5)Do you know what steps to take if you face such a problem?	Yes/No
6)Do you know the basic causes of common speech delays and disorders among children?	Yes/No
7)Do you know that such problems should be addressed soon?	Yes/No
8) Did you find this awareness study useful?	Yes/No
